# Trans-hiatal herniation following esophagectomy or gastrectomy: retrospective single-center experiences with a potential surgical emergency

**DOI:** 10.1007/s10029-021-02380-1

**Published:** 2021-03-13

**Authors:** P. U. Oppelt, I. Askevold, R. Hörbelt, F. C. Roller, W. Padberg, A. Hecker, M. Reichert

**Affiliations:** 1 Department of General, Visceral, Thoracic, Transplant and Pediatric Surgery, University Hospital of Giessen, Rudolf-Buchheim-Strasse 7, 35392 Giessen, Germany; 2 Department of Diagnostic and Interventional Radiology, University Hospital of Giessen, Klinikstrasse 33, 35392 Giessen, Germany

**Keywords:** Trans-hiatal herniation, Trans-hiatal hernia, Esophagectomy, Gastrectomy, Hiatal hernia repair, Emergency surgery

## Abstract

**Purpose:**

Trans-hiatal herniation after esophago-gastric surgery is a potentially severe complication due to the risk of bowel incarceration and cardiac or respiratory complaints. However, measures for prevention and treatment options are based on a single surgeon´s experiences and small case series in the literature.

**Methods:**

Retrospective single-center analysis on patients who underwent surgical repair of trans-hiatal hernia following gastrectomy or esophagectomy from 01/2003 to 07/2020 regarding clinical symptoms, hernia characteristics, pre-operative imaging, hernia repair technique and perioperative outcome.

**Results:**

Trans-hiatal hernia repair was performed in 9 patients following abdomino-thoracic esophagectomy (40.9%), in 8 patients following trans-hiatal esophagectomy (36.4%) and in 5 patients following conventional gastrectomy (22.7%). Gastrointestinal symptoms with bowel obstruction and pain were mostly prevalent (63.6 and 59.1%, respectively), two patients were asymptomatic. Transverse colon (54.5%) and small intestine (77.3%) most frequently prolapsed into the left chest after esophagectomy (88.2%) and into the dorsal mediastinum after gastrectomy (60.0%). Half of the patients had signs of incarceration in pre-operative imaging, 10 patients underwent emergency surgery. However, bowel resection was only necessary in one patient. Hernia repair was performed by suture cruroplasty without (*n = *12) or with mesh reinforcement (*n = *5) or tension-free mesh interposition (*n = *5). Postoperative pleural complications were most frequently observed, especially in patients who underwent any kind of mesh repair. Three patients developed recurrency, of whom two underwent again surgical repair.

**Conclusion:**

Trans-hiatal herniation after esophago-gastric surgery is rare but relevant. The role of surgical repair in asymptomatic patients is disputed. However, early hernia repair prevents patients from severe complications. Measures for prevention and adequate closure techniques are not yet defined.

## Introduction

Esophago-gastric surgery bears an extraordinary high risk for postoperative complications, which consecutively contribute majorly to postoperative morbidity and mortality even in high-volume, well-experienced operative centers [1, 2]. Vice versa, postoperative complications not only impair short- but also (oncologic) long-term outcomes of the affected patients [1–3]. Especially pulmonary complications, including pneumonia, respiratory failure and respiratory distress, as well as anastomotic complications, predominantly leakages, become critically apparent immediately after surgery [4–6]. Furthermore, anastomotic stenosis, reflux disease, malnutrition and even tumor recurrencies after oncologic esophagectomy or gastrectomy are well-known clinical problems in the long-run after surgery. Nevertheless, these complex surgical approaches for esophagectomy or gastrectomy bear the risk for some other, rather uncommon surgical complications, which might appear during both the short-term as well as long-term follow-up of the patients. Thereby, it is well known that an early and correct diagnosis as well as early and appropriate therapy of severe and potentially life-threatening complications after esophago-gastric surgery, reduce further morbidity and mortality of the patients and is one of the main differences between the high-volume and lower-volume centers for upper gastrointestinal surgery [1, 2]. One of these rare but feared complications is trans-hiatal herniation of abdominal viscera following gastrectomy or esophagectomy [7]. Already in 2016 Crespin and colleagues entitled post-esophagectomy hiatal hernia (HH) as “an underreported complication” with a cumulative incidence even in asymptomatic patients being up to 26% [8, 9]. HH after esophago-gastric surgery might cause severe, life-threatening complications and critical illness in a high percentage of the affected symptomatic patients [7, 10]. However, clear evidence for strategies on “how to approach the hiatus” during the initial surgery to prevent patients from trans-hiatal herniation following esophago-gastric surgery are not yet established [7, 8, 10–22]. Furthermore, symptoms of the patients reported in mainly small case series in the literature vary strongly [7, 16] and, although surgery is the only therapeutic solution, guidelines or recommendations from the respective medical societies for adequate diagnosis and surgical hernia repair addressing the question “when, why and how” are currently missing [7]. Thus, the knowledge about symptoms, adequate diagnosis and imaging techniques as well as appropriate surgical treatment is only based on a single surgeon’s experiences as well as case reports and small retrospective case series from the current literature without providing sufficient long-term follow-up [23]. Questions may arise, when and why to operate even asymptomatic patients with an incidental finding of trans-hiatal herniation after esophago-gastric surgery considering the high risk for incarceration, how to perform the surgery for hernia repair and by which technique to close the hiatal defect considering the size and the consecutive risk for recurrency: suture cruroplasty, additional mesh augmentation or tension-free mesh interposition? Therefore, it seems to be important to improve the evidence by providing single institutional experiences along with our patient cohort and presenting a systematic review of case series on trans-hiatal hernia repair after esophago-gastric surgery from the current literature.

## Materials and methods

### Patients and study design

We retrospectively evaluated adult patients who underwent repair of trans-hiatal hernia from 01/2003 to 07/2020 at the University Hospital of Giessen with a special focus on patients, who were operated on HH following previous esophago-gastric surgery. The retrospective data acquisition was formally approved by the local ethics committee of the medical faculty of the University of Giessen (approval numbers: 214/15, 253/16 and 97/19). Each patient was treated by the local standard-of-care.

Inclusion criteria were adult patients (≥ 18 years of age) with trans-hiatal herniation (i.e. prolapse of abdominal contents into the chest cavities or the posterior mediastinum) following gastrectomy, trans-hiatal esophagectomy or abdomino-thoracic esophagectomy in the patients´ history. Other types of trans-diaphragmatic hernia and even re-do surgeries for recurrent HH without previous gastrectomy and/or esophagectomy as well as HH after bariatric surgery were excluded from the analysis.

Outcome parameters were symptoms of the patients, preoperative diagnostic modalities and findings, indication for surgery, character of surgery (emergency versus elective), surgical technique and modalities of hernia repair (primary suture with or without mesh augmentation and mesh interposition), duration of surgery and postoperative stay on intensive care unit (ICU), total postoperative in-hospital stay as well as postoperative complications. The latter were assessed during the postoperative in-hospital stay or during the initial 30 postoperative days. Patient data and patient characteristics were evaluated retrospectively from the prospectively maintained institutional database.

**Fig. 1:**
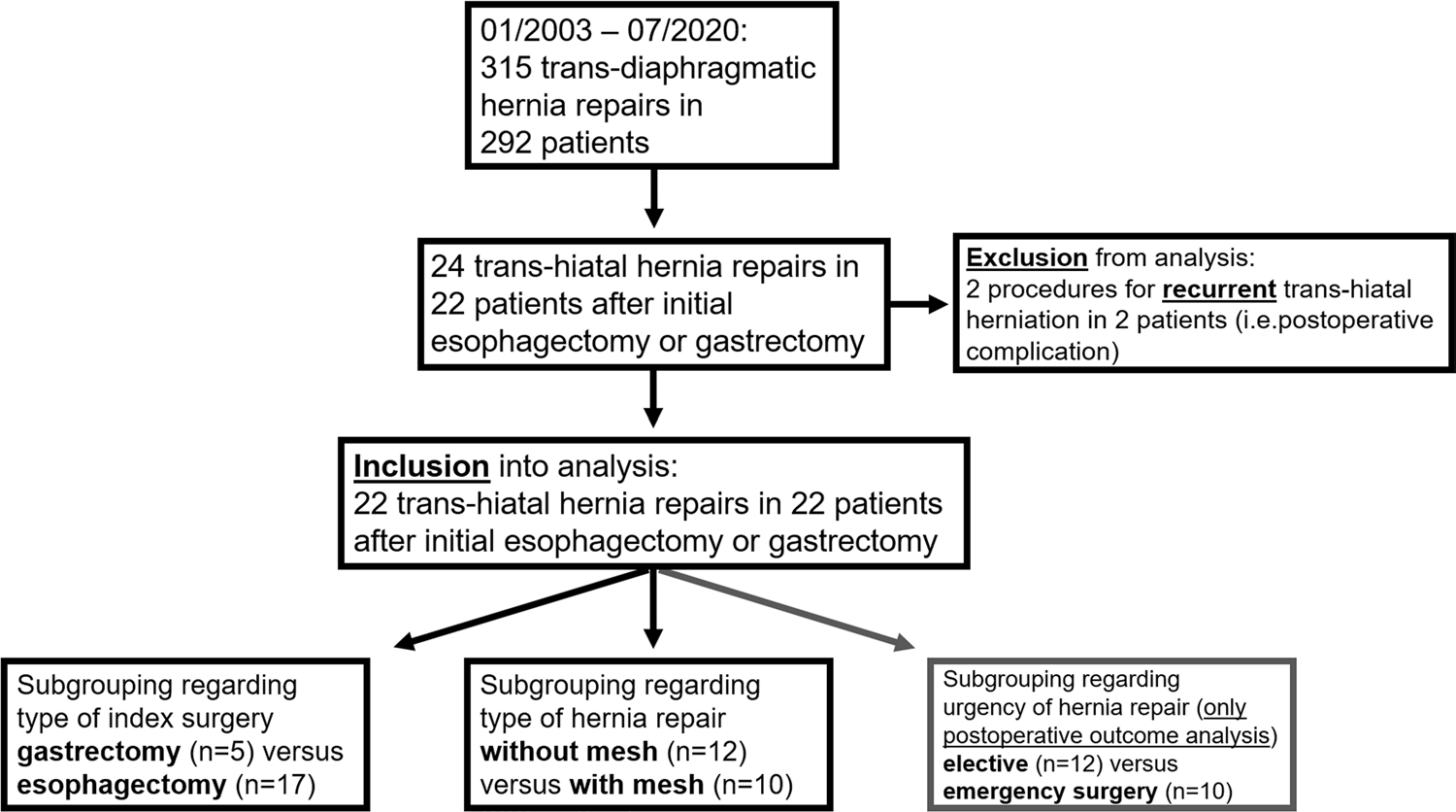
Patient cohort and subgrouping

After a primary analysis of patient data, patients were divided into one group who initially had undergone gastrectomy and another group who initially had undergone esophagectomy (including both trans-hiatal and abdomino-thoracic esophagectomy) to compare differences by the initial surgical procedure. Secondly, the patients were divided according to the surgical closure technique of the HH: patients who underwent either “only” primary suture for hernia repair or patients who underwent mesh-repair, including mesh augmentation (after suture cruroplasty) or tension-free mesh interposition. Results of both analyses are presented in the tables. Furthermore, for a better understanding of therapeutic decision making and perioperative patient outcome evaluation, postoperative outcome parameters were compared regarding the urgency of surgery: elective versus emergency hernia repair (Fig. 1 gives an overview of the patient cohort and subgrouping). Statistical analyses were performed using GraphPad Prism (Version 5.00 for Windows, GraphPad Software, San Diego California USA, www.graphpad.com). Two-group comparisons (regarding initial surgery: gastrectomy versus esophagectomy, hernia closure technique: with versus without mesh, or the urgency of hernia repair: elective versus emergency) were analyzed using Mann–Whitney-U test for continuous data or Pearson’s *X*^2^ test for categorical data in cross-tabulation. Data are given as *n* (%) or medians and ranges (i.e. minimum–maximum); *p *values ≤ 0.05 were considered to indicate statistical significance.

### Surgical technique

Basically, the institutional surgical techniques approaching diaphragmatic hernia had been described previously [24]. Abdominal approaches play the leading role for HH repair after esophago-gastric surgery. Patients who undergo open surgical hernia repair are placed in the supine position and a median laparotomy is used to access the epigastric region under general anesthesia. Hernia contents are gently reduced and retrieved into the abdominal cavity as the first step. In cases of classical HH repair, excision and resection of the hernia sac is the standard procedure in our institution, however, there is usually no hernia sac present surrounding the prolapsed organs in cases of trans-hiatal hernia after esophago-gastric surgery [10]. The hiatal defect is closed by cruroplasty primarily with thick, non-resorbable, interrupted sutures. If a mesh is used to buttress the cruroplasty, the size of the mesh is considered to overlap the repair site approximately 5 cm beyond the edges of the defect. Single sutures or endo-staplers are used for the fixation of the mesh. If primary cruroplasty is not possible, due to the large size of the defect, a mesh interposition is inserted anteriorly from the diaphragm to the gastric conduit after esophagectomy or the jejunal limb to the anastomosis after trans-hiatal esophagectomy with gastrectomy, thus the conduit is located dorsally.

In laparoscopic, minimally invasive surgery for HH repair we basically follow the same principles as in open surgery. Here, patients are placed in reverse Trendelenburg position. The mesh for reinforcement of the cruroplasty is fixed in laparoscopic surgery by using endo-stapling devices. The institutional operation technique for laparoscopic hernia repair with mesh augmentation of trans-hiatal herniation after esophago-gastric surgery is adopted from the clinical standard of classical HH repair.

### Literature review

To improve the evidence and the discussion of manuscript data, a systematic literature review on the repair of trans-hiatal hernia following gastrectomy, trans-hiatal or abdomino-thoracic (-cervical) esophagectomy was performed. English literature in Medline was systematically searched in September 2020. Original case series published from 01/2005 to 09/2020 containing ≥ 5 adult cases, who underwent surgery for trans-hiatal hernia repair after an initial esophagectomy or gastrectomy were included. Reports on HH repair following esophago-gastric resections containing < 5 surgical procedures, para-hiatal hernia or HH repair following bariatric surgery including gastric bypass or sleeve gastrectomy were excluded from the literature review.

## Results

### General characteristics of the patient cohort

Between 01/2003 and 07/2020 three-hundred-fifteen surgical procedures were performed in 292 patients for any type of trans-diaphragmatic hernia. Amongst them, 24 operations, i.e. 7.6% of the total procedures, were performed for trans-hiatal hernia in 22 patients following abdomino-thoracic esophagectomy (*n = *9, 40.9%), trans-hiatal esophagectomy (*n = *8, 36.4%) or conventional gastrectomy (*n = *5, 22.7%) and met the inclusion criteria. The remaining 2 procedures in 2 patients were performed for repeated repair of recurrent post-esophagectomy trans-hiatal herniation and were classified as a short- or long-term complication, respectively, in the patient outcome analysis of the present study (Fig. 1). The patients, included in this study, had undergone index surgeries between the years 1998 and 2018; twenty of the patients had undergone esophagectomy or gastrectomy initially at our institution, thus detailed information on the initial procedure was available. All patients of the esophagectomy group initially had undergone resection for oncologic purposes, including carcinoma of the thoracic esophagus or gastro-esophageal junction (GEJ) in 16 of 17 patients. Among them, conventional open trans-hiatal esophagectomy with gastrectomy and Roux-Y reconstruction had been performed in 7 patients and trans-hiatal esophagectomy with fundectomy and gastric conduit reconstruction had been performed in one patient. In the remaining 9 patients abdomino-thoracic esophagectomy had been performed with gastric conduit for reconstruction (conventional open surgery in 6 patients and a hybrid minimally invasive, laparoscopically assisted approach in 3 patients). Two patients from the gastrectomy group initially had undergone oncologic resection for gastric carcinoma, three patients initially had undergone gastrectomy for incarcerated paraesophageal hernia. Notably, in 9 patients of the esophagectomy group, especially in those with a carcinoma of the GEJ, resection of diaphragmatic crura had been performed during initial surgery versus in none of the gastrectomy patients (*p* = 0.03, Table 1). The size of the hernial orifice was extensively in patients with crus resection during index surgery [9.7 (6.7–12.8) cm versus patients without crus resection: 6.8 (4.4–9.7) cm, *p* < 0.01]. All of the patients, who suffered from trans-hiatal prolapse of abdominal contents into the chest immediately [i.e. in median 6 (6–13) days] after index surgery, underwent resection of diaphragmatic crura during the initial trans-hiatal (*n = *3) or abdomino-thoracic esophagectomy (*n = *2). Two of these patients who developed the hernia early post-esophagectomy suffered from herniation into both sides of the chest. Clinically, three patients with developing the hernia immediately suffered from severe cardiac (*n = *2: tachyarrythmia and *n = *1: cardiac arrest) and respiratory symptoms (*n = *2) prior to hernia repair. Bowel incarceration was detected in two of these patients by preoperative computed tomography (CT). Four of these patients underwent emergency surgery. Hernia were repaired by simple sutures without mesh reinforcement in all five patients.

**Table 1:** Patient characteristics

Variables	All patients	Subgroup analysis					
	(*n = *22)	Initial surgery			Mesh repair		
		Gastrectomy	Esophagectomy	*p*-value	Without mesh	With mesh	*p*-value
		(*n = *5)	(*n = *17)		(*n = *12)	(*n = *10)	
Male gender	16	3	13	0.47	9	7	0.79
Age (years)	66.5 (26–81)	69 (26–78)	65 (45–81)	0.94	64 (26–79)	72 (48–81)	0.2
Body mass index (kg/m^2^)	21.6 (15.8–34.7)	22.0 (15.8–29.4)	21.1 (18.0–34.7)	0.85	21.6 (15.8–34.7)	22.5 (19.0–29.4)	0.58
Chronic diseases	17	3	14	0.29	9	8	0.78
Cardiac	12	1	11		6	6	
Pulmonal	6	1	5		4	2	
Renal	1	1	0		0	1	
Symptoms							
None	2	1	1	0.33	1	1	0.89
Dyspnoe	5	1	4	0.87	3	2	0.78
Cardiac	4^#^	0	4	0.23	4	0	0.04
Gastrointestinal	14	4	10	0.37	7	7	0.57
Ileus	7	2	5	0.66	3	4	0.54
Dysphagia	3	0	3	0.31	0	3	0.04
Reflux disease*	2	1	1	0.33	1	1	0.89
Pain	13	3	10	0.96	7	6	0.94
Thoracic	4	1	3	0.9	2	2	0.84
Abdominal	11	3	8	0.61	7	4	0.39
Characteristics of initial surgery							
Oncologic indication	19	2 ^ß^	17	< 0.01	11	8	0.43
Conventional gastrectomy	5	5	–		3	2	0.78
Trans-hiatal esophagectomy with gastrectomy (Roux-Y reconstruction)	7	–	7		4	3	
Trans-hiatal esophagectomy (with gastric conduit reconstruction)	1	–	1		1	0	
Abdomino-thoracic esophagectomy (with gastric conduit reconstruction)	9	–	9		4	5	
Additional procedures during initial surgery [n patients]^¶^	8	2	6	0.85	6	2	0.15
Minor surgery [n procedures]	9	1	8		7	2	
Major surgery [n procedures]	4	2^§^	2^&^		3^@^	1 €	
Crus resection during initial surgery [n patients]^¶^	9	0	9	0.03	7	2	0.07
Comprehensive complication index after initial surgery^µ^	34.6 (0–99.9)	35.9 (22.6–47.4)	33.5 (0–99.9)	0.73	39.5 (0–99.9)	21.1 (0–54.2)	0.43
Duration from initial surgery to							
Hernia diagnosis [d]	320 (6–3884)	748 (285–2624)	143 (6–3884)	0.1	81 (6–2624)	619.5 (143–3884)	0.01
Hernia surgery [d]	368 (6–3891)	751 (289–2650)	171.5 (6–3891)	0.15	81 (6–2650)	751 (242–3891)	0.01
Duration of symptoms or time from diagnosis to hernia surgery ≤ 1 day [n patients]	10	2	8	0.78	8	2	0.03
Elective surgery	12	3	9	0.78	4	8	0.03
Emergency surgery	10	2	8		8	2	

^*^ Including recurrent acid aspiration in both patients. # including cardiopulmonary resuscitation in 1 case

§ Splenectomy in both cases

& Including resection of the right upper pulmonary lobe in one case and laryngectomy in one case

¶ Unknown in one case with an initial trans-hiatal esophagectomy with gastrectomy from the group of patients who underwent mesh repair of the hernia.

€ Including the laryngectomy

@ Including splenectomy in two cases and resection of the right upper pulmonary lobe in one case

ß Three gastrectomies performed for incarcerated up-side down stomach or paraesophageal hernia. µ postoperative complications after index surgery were not available retrospectively in 3 patients (one from the gastrectomy and two from the esophagectomy group as well as one from the mesh- and two from the mesh + group, respectively); please be aware, that trans-hiatal hernia occurred immediately after index surgery (during the initial hospital stay or initial 30 postoperative days) in 5 patients (all from the esophagectomy group)

In one patient, who initially had undergone an abdomino-thoracic esophagectomy, the hernia was electively approached by laparoscopy (Table 1). Thereby, the contents of the hernia (intraoperatively assessed in this case: transverse colon and omentum majus) were reduced and the orifice was closed by single suture cruroplasty with mesh augmentation. Of note, signs of incarceration, including bowel obstruction or vascular strangulation, were not detected in the preoperative CT in this case (Table 2). One patient after an initial abdomino-thoracic esophagectomy underwent again an abdomino-thoracic approach for hernia repair with left-sided antero-lateral thoracotomy for lysis of the hernia contents out of the left chest (herniation of transverse colon and subtotal herniation of the small bowel). The large hiatal defect was closed in this case with mesh interposition during the abdominal part of the surgery.

**Table 2:** Hernia characteristics

Variables	All patients (*n = *22)		Subgroup analysis											
			Initial surgery						Mesh repair					
			Gastrectomy		Esophagectomy		*p*-value		Without mesh		With mesh		*p*-value	
			(*n = *5)		(*n = *17)				(*n = *12)		(*n = *10)			
Localization							0.02						0.61	
Dorsal mediastinum, i.e. “axial”	4		3		1				2		2			
Left-sided enterothorax	14		1		13				7		7			
Right-sided enterothorax	2		1		1				1		1			
Both-sided enterothorax	2		0		2				2		0			
Imaging^$^: Contents of the hernia														
Transverse colon	10		1		9^#^		0.19		5^#^		5		0.7	
Small intestine^§^	15		4		11		0.52		9		6		0.45	
Pancreas*	2		0		2		0.42		0		2		0.1	
Liver	1		0		1		0.58		1		0		0.35	
Omentum majus	3		0		3		0.31		0		3		0.04	
CT: Signs of incarceration	11		3		8		0.61		8		3		0.09	
Bowel obstruction/ileus	11		3		8		0.61		8		3		0.09	
Vascular strangulation	7		2		5		0.66		5		2		0.28	
Ileus + vascular strangulation	7		2		5		0.66		5		2		0.28	
IO: Contents of the hernia														
Transverse colon	12		0		12^ß^		0.01		7		5		0.7	
Small intestine	17		5		12		0.17		9		8		0.78	
Esophagojejunostomy	2		2		–				1		1		0.89	
Pancreas*	1		0		1		0.58		0		1		0.26	
Liver	0		0		0		1		0		0		1	
Omentum majus	2		0		2		0.42		0		2		0.1	
IO														
Signs of vascular strangulation	5		1		4		0.87		4		1		0.19	
Bowel ischemia^€^	1		0		1		0.58		1		0		0.35	
Bowel fixation/pexy of abdominal contents	5		3		2		0.02		5		0		0.02	
Hernia closure technique														
Primary suture cruroplasty	12		3		9		0.78		12		–			
Mesh augmentation^¶^	5		2		3		0.29		–		5			
Mesh interposition	5		0		5		0.17		–		5			
Lateral diameter of the hernial orifice in pre-operative CT-scan [cm]^&^	7.9 (4.4–12.8)		5.6 (4.4–9.4)		9.3 (6.0–12.8)		0.04		8.7 (4.4–12.0)		7.7 (6.0–12.8)		0.82	

*CT* Computed tomography, *IO* intraoperative

^$^ Computed tomography in 21 patients, conventional chest X-ray in 1 patient from the esophagectomy group

# Subtotal colonic herniation in 5 cases

§ Most commonly subtotal small intestine herniation

* Herniation of the pancreatic corpus and tail

¶ After suture cruroplasty. ß Subtotal colonic herniation in 3 cases

€ Irreversible ischemia with bowel gangrene and consecutive right hemicolectomy in one case

& The largest lateral diameter of the hernial orifice from both diaphragmatic crura was measured after adjusting the CT-scan by the axis of the hernia; not available in two patients from the esophagectomy/hernia repair with mesh interposition group: one patient did not receive CT-scan before surgery and in one patient the pre-operative CT-scan was not available retrospectively. No differences were observed regarding the orificial sizes from patients who underwent suture cruroplasty with or without mesh augmentation [7.9 (4.4–12.0)] versus closure with tension-free mesh interposition [8.2 (6.0–12.8), *p* = 0.67]

### Symptoms of the patients and radiographic diagnosis

Preoperative symptoms varied strongly among the patients. Gastrointestinal symptoms with bowel obstruction as well as abdominal and/or thoracic pain were mostly prevalent (63.6 as well as 59.1%, respectively). In the group of patients who underwent primary suture (without mesh repair) cardiac symptoms were more frequently prevalent (*p* = 0.04) and these patients underwent more frequently emergency surgery for hernia repair (*p* = 0.03, Tables 1, 2). Notably, two patients, who suffered from severe abdominal pain, presented with serological and radiological signs of acute pancreatitis as well as signs of cholestasis. In both patients the biliodigestive limb after an initial trans-hiatal esophagectomy with gastrectomy and Roux-Y reconstruction was affected and consecutively obstructed by the HH, resulting in a surgical emergency.

In 95.5% of the patients, cross-sectional imaging with CT was sufficient for preoperative diagnosis of trans-hiatal herniation after esophago-gastric surgery. In one single case pre-operative imaging was limited to conventional chest X-ray, which confirmed extensive small bowel herniation into the left chest (Table 2). Additional diagnostic tools played a minor role in the preoperative work-up. Therefore, endoscopy (*n = *4) and barium swallow imaging study (*n = *1) were applied for a more functional evaluation of the upper gastrointestinal tract, e.g. for diagnosis of reflux and dysphagia (both in two different patients) or to exclude local recurrencies after an initial oncologic esophagectomy or gastrectomy. These “extended” pre-operative diagnostic work-up did not play a role in the emergency setting; all of these five patients with an “extended” pre-operative diagnostic work-up underwent elective surgery for hernia repair.

### Hernia repair

There were some discrepancies between the localization of the herniation between patients, who initially had undergone a gastrectomy versus esophagectomy: whereas patients after gastrectomy suffered more frequently from an “axial” herniation into the posterior mediastinum (60%) containing parts of the small bowel, patients after esophagectomy suffered most commonly from an extensive trans-hiatal herniation into the left thoracic cavity (76.5%) or both sides (11.8%). Small bowel (in most cases subtotal proportion of the small bowel) and transverse colon most commonly prolapsed into the chest, but also the pancreatic tail and parts of the liver were found as hernia contents in preoperative CT. However, some  discrepancies between preoperative imaging by CT and intraoperative findings during hernia repair concerning the hernia contents as well as sings of incarceration were observed, as shown in Table 2. After reducing the hernia contents gently into the abdominal cavity, the orifice of the hernia was repaired in 12 patients by suture cruroplasty without the usage of meshes. In the remaining 10 patients, meshes were used for hernia repair (*n = *5: mesh augmentation after suture cruroplasty and *n = *5: tension-free mesh interposition). In patients without the application of meshes abdominal viscera (especially transverse colon) were fixed surgically by sutures more frequently in the abdominal cavity to prevent re-herniation compared with patients who underwent any kind of mesh repair (*p* = 0.02, Table 2). Interestingly, patients underwent more frequent hernia repair by simple suture cruroplasty without mesh augmentation or mesh interposition during emergency surgery (*p* = 0.03, Table 1).

### Perioperative outcome

Higher peripheral blood leucocytes and serum C-reactive protein values prior to hernia repair might be an expression of the emergency character due to bowel incarceration in the group of patients, who underwent surgery without mesh implantation (Table 3). Basically, differences in postoperative inflammatory markers as indicators for surgical trauma or infectious complications as well as in the overall incidence for severe postoperative complications (≥ grade 3 concerning the Clavien-Dindo classification of surgical complications [25]) were not observed after hernia repair with regard to index surgery (≥ grade 3 after initial gastrectomy versus esophagectomy: *p* = 0.61), usage of meshes (≥ grade 3 after hiatal hernia repair with versus without mesh: *p* = 0.39, Table 4) or the urgency of hernia repair (≥ grade 3 after emergency versus elective surgery for hernia repair: *p* = 1). However, a pleural fluid drainage was placed in seven patients (all from the group of patients, who initially had undergone esophagectomy) at the end of hernia repair surgery into the affected thoracic cavity. Postoperatively, pleural complications including recurrent pleural fluid collections and re-drainage of the thoracic cavity were the most frequently observed complications. Although the number of patients, who developed recurrent pleural fluid collections, was not significantly different between the subgroups (*p* = 0.09), pleural re-drainage was necessary more frequently in patients after mesh repair of the hernia (*p* = 0.01, Table 4)—regardless of the urgency of surgery (*p* = 0.28). Furthermore, two patients from the esophagectomy group suffered from severe or fatal complications, indicated by an extraordinary high comprehensive complication index [26]. One of these patients died on the 12th postoperative day due to cerebral hemorrhage after infarction following cardiac arrest immediately after hernia repair. Three patients developed recurrency: two of them underwent again surgical repair for recurrent trans-hiatal hernia (Table 4).

**Table 3:** Perioperative inflammatory marker profile

Variables	All patients (*n = *22)	Subgroup analysis					
		Initial surgery			Mesh augmentation		
		Gastrectomy	Esophagectomy	*p*-value	Without mesh	With mesh	*p*-value
		(*n = *5)	(*n = *17)		(*n = *12)	(*n = *10)	
Perioperative leucocytes [giga/l]							
Preoperative	8.3 (3.7–26.5)	9.5 (3.7–20.3)	7.9 (4.7–26.5)	0.97	9.6 (5.2–26.5)	6.2 (3.7–12.6)	0.04
POD 1	11.2 (0.1–30.5)^#^	12.2 (9.6–18.5)	11.2 (0.1–30.5)^#^	0.46	11.4 (0.1–30.5)	11.2 (8.1–18.5)^#^	0.62
POD 3–5*	8.4 (4.6–27.3)	8.1 (6.3–15.6)	8.6 (4.6–27.3)	0.97	10.6 (4.6–27.3)	8.0 (6.3–16.1)	0.6
POD 5–15*	9.7 (3.2–59.2)^#^	9.7 (6.3–15.1)	10.5 (3.2–59.2)^#^	0.8	10.4 (3.2–59.2)	9.7 (5.1–14.6)^#^	0.48
Perioperative CRP [mg/l]							
Preoperative	6.3 (0.5–156.0)	6.9 (0.8–80.6)	5.7 (0.5–156.0)	0.88	49.3 (0.5–156.0)	1.5 (0.5–71.2)	0.04
POD 1	68.5 (0.8–182.3)	48.0 (9.2–92.5)	72.9 (0.8–182.3)	0.24	78.9 (0.8–182.3)	67.7 (11.6–82.7)	0.49
POD 3–5*	119.0 (37.4–479.1)	81.2 (43.6–160.8)	152.2 (37.4–479.1)	0.09	138.1 (68.6–269.5)	110.2 (37.4–479.1)	0.53
POD 5–15*	77.5 (17.3–585.6) ^#^	61.2 (22.0–114.0)	89.4 (17.3–585.6)^#^	0.34	101.7 (22.0–246.6)	96.4 (17.3–585.6)^#^	0.17

*POD* postoperative day, *CRP* C-reactive protein

^*^ Highest value at postoperative day 3–5 or 5–15, respectively

# Not available in one patient retrospectively

**Table 4:** Perioperative outcome

Variables	All patients (*n = *22)	Subgroup analysis					
		Initial surgery			Mesh augmentation		
		Gastrectomy	Esophagectomy	*p*-value	Without mesh	With mesh	*p*-value
		(*n = *5)	(*n = *17)		(*n = *12)	(*n = *10)	
Duration of hernia surgery [min]	103 (47–461)	152 (47–239)	102 (47–461)	0.97	95.5 (47–276)	129 (66–461)	0.12
Postoperative in-hospital stay [d]							
ICU	2.5 (0–157)	1 (0–12)	3 (0–157)	0.14	3 (0–157)	1.5 (0–12)	0.55
Total	9.5 (4–157)	8 (6–20)	10 (4–157)	0.58	10 (6–157)	8.5 (4–20)	0.49
Postoperative complications^§^							
Comprehensive complication index	23.56 (0–43.8)	20.9 (8.7–42.6)	33.5 (0–100)	0.69	20.9 (0–99.9)	42.6 (0–100)	0.49
≥ Grade 3*	11	2	9	0.61	5	6	0.39
Recurrent pleural fluid collections	11	2	9	0.61	4	7	0.09
Pleural re-drainage	7	1	6	0.52	1	6	0.01
Recurrency of hernia	3	1^$^	2^¶^	0.63	1^$^	2^¶^	0.43

*ICU* Intensive care unit

^§^ Complications during the postoperative hospital stay or during the initial 30 postoperative days after the hernia repair * regarding the Clavien-Dindo classification of surgical complications [25] as well as expressed by the comprehensive complication index [26]

$ recurrent trans-hiatal hernia into the dorsal mediastinum occurred after an initial gastrectomy in the long-run (7 months) after suture cruroplasty and was decided for non-operative management due to the absence of symptoms and worse clinical condition of the patient

¶ Two patients after initial esophagectomy (one trans-hiatal, one abdomino-thoracic), who underwent mesh repair (one augmentation and one interposition). Both patients underwent surgery for the recurrent hernia again: one patient after an initial abdomino-thoracic esophagectomy with resection of the diaphragmatic crura and left-sided trans-hiatal enterothorax, who underwent mesh interposition due to a large hiatal orifice two years after index surgery, developed hernia recurrency on postoperative day one due to tear out of the mesh with consecutive surgical revision. The other patient underwent suture cruroplasty with mesh augmentation for trans-hiatal herniation into the left chest 10 years after trans-hiatal esophagectomy and developed symptomatic recurrency of the hernia five months later with consecutive hernia repair by suture cruroplasty

## Discussion

Esophagectomy and gastrectomy, especially for cancer, are high-risk procedures bearing the potential for postoperative severe and life-threatening complications [1–3]. Short-term outcome, lengths of hospital stay and health care costs are determined by the development of postoperative, especially pulmonary, cardiac and anastomotic complications [1–6, 27]. Severe complications, thereby, have not only the potential to dramatically increase early postoperative mortality rate but also to impair even long-term and oncological outcomes, thus an early recognition and adequate therapy of the complication is mandatory for the affected patients [1–3]. A currently underreported complication is the development of trans-hiatal herniation, which can occur during both the short- and long-term follow-up after esophago-gastric surgery [7, 8, 10, 17, 28]. Therefore, Oor et al. described in their meta-analysis from 2016 lower pooled incidences of HH being 1.0% after conventional open and quite higher (4.5%) after minimally-invasive esophagectomy [29], which might be dramatically underestimated since structured upper gastrointestinal cancer surveillance with cross-sectional imaging especially in patients without symptoms is almost lacking [8, 30, 31]. Furthermore, (asymptomatic) patients who underwent non-operative management of HH after esophagectomy are underreported within predominantly surgical articles (Table 5). But, most importantly, only the vast minority of post-esophagectomy HH were reported initially by radiologists upon the CT scans [8, 30, 31]. Thus, in the very recent literature some authors depicted the “true” incidence of post-esophagectomy HH beeing unequally higher (up to 26% after minimally invasive esophagectomy) [8–10, 17, 19, 28]. However, as proven by our presented data as well as literature review in Table 5, only little experience with trans-hiatal hernia repair after esophagectomy or gastrectomy exists even in higher-volume centers for upper gastrointestinal surgery.

**Table 5:** Systematic literature review on trans-hiatal herniation following gastrectomy and esophagectomy

Author [Reference number]	Reference	Study information, Observational period	General characteristics of the study cohort, *n* patients	Symptoms, Most commonly observed hernia content, Surgical hernia closure technique
Vallböhmer et al. [21]	Diaphragmatic Hernia After Conventional or Laparoscopic-Assisted Transthoracic Esophagectomy	Retrospective, Monocentric, 1997–2007	355 trans-thoracic esophagectomies, 9 (2.5%) patients were diagnosed with HH 8 (0.3–30) months after index surgery, 7 patients underwent HH repair	Asymptomatic: *n = *2 (of the operated patients) Colon + small bowel: all patients Emergency surgery: *n* = 5 Laparotomy: all patients **Type of repair:** Suture repair: *n = *6 Mesh repair: *n = *1* Recurrency: n/a
Kent et al. [42]	Revisional Surgery After Esophagectomy: An Analysis of 43 Patients	Retrospective, Monocentric, 1995–2007	24 patients developed HH *n = *7 after open trans-hiatal esophagectomy *n = *1 after open ILE *n = *2 after MI-ILE *n = *14 after MIE with cervical anastomosis 32 months (46 days-7 years) after index surgery, 22 patients underwent HH repair	Asymptomatic: *n = *4 (of all patients) Colon: *n = *92% Small bowel: *n = *21% Emergency surgery: *n* = 4 Laparotomy: *n = *7 (conversion: *n = *2) Laparoscopy: *n = *15 **Type of repair:** Suture repair (+ Pexy of the conduit to the diaphragm): *n = *22 Direct suture + mesh reinforcement: *n = *9 Recurrency: *n = *6
Sutherland et al. [37]	Postoperative incidence of incarcerated hiatal hernia and its prevention after robotic transhiatal esophagectomy	Retrospective, Monocentric, 2007–2009	36 trans-hiatal robot-assisted total esophagectomies with gastric conduit and cervical anastomosis, 7 (19.4%) patients underwent HH repair for incarceration: < POD 30: *n = *1 POD 30–60: *n = *1 POD 60–120: *n = *3 > POD 120: *n = *2	Asymptomatic: *n = *0 Most common hernia content: n/a Emergency surgery: *n = *6 Surgical approach: n/a **Type of repair:** Direct suture + mesh reinforcement: *n = *7 Recurrency: *n = *2
Price et al. [40]	Hiatal Hernia After Esophagectomy: Analysis of 2,182 Esophagectomies From a Singe Institution	Retrospective, Monocentric 1988–2008	2182 esophagectomies, 15 (0.69%) patients developed HH and underwent hernia repair 1 year 9 months (3 days – 12 years) after index surgery *n* = 9 (0.92%) after ILE *n* = 5 (0.83%) after trans-hiatal esophagectomy *n* = 1 after substernal colonic interposition	Asymptomatic: *n = *2 Colon: *n = *6 Colon + small bowel: *n = *4 Small bowel: *n = *5 Emergency surgery: *n = *2 Trans-abdominal approach: *n = *14 Laparotomy/Laparoscopy: n/a Trans-thoracic approach: *n = *1 **Type of repair:** Suture repair: *n = *13 Mesh repair: *n = *2* Recurrency: *n = *2
Erkmen et al. [18]	Laparoscopic repair of Hiatal Hernia After Esophagectomy	Retrospective, Monocentric, 2011–2017	Total number of esophagectomies: n/a 5 patients underwent surgery for intended hiatal repair* 24 (10–36) months post-esophagectomy ^#^ in one patient with carcinomatosis diagnosed during laparoscopy hernia repair was aborted!	Asymptomatic: *n = *0 Colon: *n = *2 Colon + small bowel: *n = *2 Small bowel: *n = *0 Emergency surgery: n/a Laparoscopy: *n = *5^#^ **Type of repair**^**#**^**:** Direct suture: *n = *3 Mesh interposition: *n = *1 Recurrency: *n = *1
Ganeshan et al [30]	Diaphragmatic Herniation After Esophagectomy in 440 Patients With Long-Term Follow-up	Retrospective, Monocentric, 2001–2007	440 esophagectomies, retrospective assessment of CT scans 67 (15%) patients developed HH 2 years (47 days–9.35 years) after esophagectomy *n = *32 (12%) after ILE *n = *7 (17%) after McKewon esophagectomy *n = *25 (24%) after trans-hiatal esophagectomy *n = *3 (10%) after MIE 9 patients underwent HH repair	Asymptomatic: *n = *2 (of the operated patients) Colon: *n = *28 (all patients; *n = *6 of the operated patients) Colon + small bowel: *n = *4 (all patients; *n = *2 of the operated patients) Small bowel: *n = *1 (all / operated patient) Emergency surgery: *n* = 5 Laparotomy: *n = *8 Laparoscopy: *n = *1 **Type of repair:** n/a Recurrency: *n = *4
Bronson et al. [16]	The Incidence of Hiatal Hernia after Minimally Invasive Esophagectomy	Retrospective, Monocentric, 2003–2011	114 MIE, 9 (8%) patients developed hernia 13.7 (1.8–55.6) months after MIE and underwent repair *n = *2 after trans-hiatal MIE *n = *7 after McKeown MIE	Asymptomatic: *n = *5 Colon: *n = *5 Colon + small bowel: *n = *2 Small bowel: *n = *1 Emergency surgery: *n = *2 Laparotomy: *n = *2 Laparoscopy: *n = *7 **Principles of repair** in all patients *: Reclosure of the crura, Biologic mesh placement and Colopexy Recurrency: *n = *1
Narayanan et al. [23]	Treatment of Diaphragmatic Hernia Occurring After Transhiatal Esophagectomy	Retrospective, Monocentric, 2000–2013	199 esophagectomies, 10 (5%) patients developed HH in median 2.4 years after esophagectomy and underwent repair *n = *9 after trans-hiatal esophagectomy *n = *1 after McKeown esophagectomy 1 patient excluded	Asymptomatic: *n = *3 Most common hernia content: n/a Emergency surgery: *n = *3 Laparotomy: all patients **Type of repair:** Mesh interposition: all patients Recurrency: *n = *0
Benjamin et al. [41]	Diaphragmatic hernia post-minimally invasive esophagectomy: a discussion and review of literature	Retrospective, Monocentric, 2006–2013,	120 MIE, 7 (5.8%) patients developed hernia 3.4 (1–45) months after MIE 5 patients underwent hernia repair 2 patients were asymptomatic and did not undergo hernia repair	Asymptomatic: *n = *0 (of the operated patients) Colon: *n = *6 Small bowel: *n = *1 Emergency surgery: *n = *1 Laparoscopy: all patients **Type of repair:** Direct suture (anterior cruropasty): *n = *3 Direct suture + mesh reinforcement: *n = *2 Recurrency: *n = *1
Messenger et al. [19]	Symptomatic diaphragmatic herniation following open and minimally invasive oesophagectomy: experience from a UK specialist unit	Retrospective, Monocentric, 1996–2012	273 esophagectomies (205 COS, 68 MIE), 11 patients developed HH 3.2 months (2 days–44 months) after esophagectomy and underwent hernia repair *n = *9 after MIE *n = *2 after open trans-hiatal esophagectomy	Asymptomatic: *n = *0 Colon: *n = *6 Colon + small bowel: *n = *4 Small bowel: *n = *1 Emergency surgery: *n = *5 Laparotomy: *n = *4 (conversion: *n = *1) Laparoscopy: *n = *7 **Type of repair:** Direct suture: *n = *6 Direct suture + mesh reinforcement: *n = *4 No closure, only omentopexy: *n = *1 (discrepancies between text and table in the manuscript) Recurrency: *n = *2
Crespin et al. [8]	Hiatal Herniation After Transhiatal Esophagectomy: an Underreported Complication	Retrospective, Monocentric 2004–2013	192 laparoscopic-assisted trans-hiatal esophagectomies, retrospective assessment of CT scans 22 patients developed HH (cumulative incidence over two years 14%) 7.5 months (2 days–97 months) after index surgery < POD 11: *n = *5 3–97 months: *n = *17 15 patients were asymptomatic (non-operative management), 7 patients were symptomatic and underwent HH repair	Asymptomatic: *n = *0 (of the operated patients) Most common hernia content: n/a Emergency surgery: *n = *2 Laparotomy: *n = *4 Laparoscopy: *n = *3 **Type of repair:** Suture repair: *n = *3 Mesh repair: *n = *4* Recurrency: *n = *1
Kanamori et al. [33]	Diaphragmatic herniation after thoracolaparoscopic esophagectomy for carcinoma of the esophagus: a report of six cases	Retrospective, Monocentric 2010–2014	150 total MIE (abdomino-thoracic) 6 (4%) patients developed HH 1–8 month after MIE, 5 patients were symptomatic and underwent HH repair, 1 patient was asymptomatic	Asymptomatic: *n = *0 (of the operated patients) Colon: *n = *2 Colon + small bowel: *n = *4 Emergency surgery: *n = *4 Laparotomy: *n = *4 Laparoscopy: *n = *1 **Type of repair:** Suture repair (+ pexy of the conduit to the diaphragm): *n = *5 Recurrency: *n = *1
Matthews et al. [28]	Diaphragmatic herniation following esophagogastric resectional surgery: an increasing problem with minimally invasive techniques?	Retrospective, Monocentric 2001–2015	631 esophagectomies, Hernia incidence (overall 5.5%): *n* = 4/221 (2%) after open 2 or 3 stage esophagecomy *n* = 22/212 (10%) after LAE *n* = 5/73 (7%) after total MIE *n* = 4/125 (3%) after total gastrectomy Hernia development: < POD 7: *n = *6 POD 7–90: *n = *6 POD 90–365: *n = *10 > POD 365: *n = *13 31 patients underwent hernia repair	Asymptomatic: *n = *1 Colon: *n = *18 Colon + small bowel: *n = *12 Small bowel: *n = *4 Emergency surgery: *n = *20 Laparotomy: *n = *20 (conversion: *n = *8) Laparoscopy: *n = *11 **Type of repair:** Suture repair: *n = *24 Mesh repair: *n = *7 Recurrency: *n = *7
Severino et al. [15]	Laparoscopic repair of hiatal hernia after minimally invasive esophagectomy	Retrospective, Monocentric 2000–2013	390 LAE, 32 (8.2%) patients developed HH 10 months (3 days–96 months) after LAE	Asymptomatic: *n = *10 Colon: *n = *29 Small bowel: *n = *4 Emergency surgery: *n = *6 Laparotomy: *n = *11 (conversion: *n = *6) Laparoscopy: *n = *19 Thoracoabdominal approach: *n = *1 (information for one patient missing in the manuscript) **Type of repair:** Suture repair: *n = *12 Mesh interposition: *n = *20 Recurrency: *n = *6
Andreou et al. [32]	Incidence and Risk Factors of Symptomatic Hiatal Hernia Following Resection for Gastric and Esophageal Cancer	Retrospective, Monocentric 2005–2012	471 esophagectomies and gastrectomies, 13 (2.8%) had symptomatic HH and underwent repair 15 (0.1–57) months after index surgery Incidences: 0.7% after gastrectomy 6.1% after extended gastrectomy 2.7% after trans-thoracic esophagectomy	Asymptomatic: *n = *0 Most common hernia content: n/a Emergency surgery: *n = *8 Laparotomy: *n = *13 **Type of repair:** Direct suture: *n = *10 Direct suture + mesh augmentation: *n = *3 Recurrency: n/a
Brenkman et al [14]	Hiatal Hernia After Esophagectomy for Cancer	Retrospective, Bicentric 2000–2014	657 (432 MIE, 225 COS) trans-hiatal, 2 and 3 stage esophagectomies, HH was diagnosed in 45 patients 20 (0–101) months after index surgery *n = *12 after COS *n = *33 after MIE 26 patients underwent HH repair 14 patients underwent emergency surgery at time of HH diagnosis 17 symptomatic patients of whom 10 initially underwent elective HH repair 14 asymptomatic patients with “wait-and-see” concept “Wait-and-see” successful in 19/21 patients	Asymptomatic: *n = *0 of the patients at time of surgery (*n = *14 initially) Colon: *n = *37 Small bowel: *n = *19 Emergency surgery: *n = *16 Laparotomy: *n = *17 (conversion: *n = *1) Laparoscopy: *n = *9 **Type of repair:** Suture repair: *n = *21 Direct suture + mesh augmentation: *n = *5 Recurrency: *n = *4
Gooszen et al. [11]	Incidence and Treatment of Symptomatic Diaphragmatic Hernia After Esophagectomy for Cancer	Retrospective, Monocentric 2005–2015	851 esophagectomies (345 MIE and 506 OE, both including THE, Ivor Lewis and McKeown esophagectomies) Symptomatic HH was diagnosed in 21 (2.5%) patients 172 (1–1031) days after index surgery All patients underwent HH repair: 4.3% after MIE (highest incidence with 9.4% after MI-ILE) 1.2% after OE	Asymptomatic: *n* = 0 Hernia contents not available in the manuscript Emergency surgery: *n = *71.4% Laparotomy: *n = *11 (Conversion: *n = *1) Laparoscopy: *n = *10 **Type of repair:** Direct suture: *n = *10 Direct suture + mesh reinforcement: *n = *11 Conduit fixation to the crus: all patients Recurrency: *n = *4
Gust et al. [7]	Hiatal hernia after oesophagectomy: a large European survey	Retrospective, Multicentric, 2000–2016	6608 esophagectomies (including THE, Ivor Lewis and McKeown esophagectomies), HH was diagnosed in 79 (1.2%) patients 78 patients [*n* = 49 (62%) after laparoscopy and *n* = 29 (37%) after laparotomy for the abdominal approach during index surgery, respectively] underwent hernia repair < POD 90: *n = *17 POD 90–365: *n = *21 > POD 365: *n = *41	Asymptomatic: *n = *11 Most common hernia content: n/a Emergency surgery: *n = *43 Laparotomy: *n = *35 (conversion: *n = *3) Laparoscopy: *n = *19 Thoracic approach: *n = *24 **Type of repair:** Direct suture ± mesh reinforcement: *n = *63 Mesh interposition: *n = *12 Mesh-repair (n/a): *n = * 3 Recurrency: *n = *8
Gong et al. [36]	Diaphragmatic Hernia After Totally Laparoscopic Total Gastrectomy for Gastric Cancer	Retrospective, Monocentric, 2011–2017	490 laparoscopic total gastrectomies, 8 (1.63%) patients underwent emergency surgery for hernia repair 7.3 (3.4–12.8) months after gastrectomy	Asymptomatic: *n* = 0 Most common hernia content: n/a Emergency surgery: *n = *8 Laparotomy: *n = *3 Laparoscopy: *n = *5 **Type of repair:** Direct suture: *n = *6 Mesh-repair (n/a): *n = *2 Recurrency: n/a
Urabe et al. [13]	Diaphragmatic herniation following total gastrectomy: review of the long-term experience of a tertiary institution	Retrospective, Monocentric, 1985–2013	1361 total gastrectomies, 5 patients underwent surgery for HH repair 78.1 (2.9–189.0) months after gastrectomy *n = *2 after laparoscopic gastrectomy *n = *3 after COS	Asymptomatic: *n* = 0 Colon: *n = *2 Colon + small bowel: *n = *1 Small bowel: *n = *2 Emergency surgery: *n = *4 Laparotomy: *n = *2 Laparoscopy: *n = *1 Left-thoracoabdominal approach: *n = *2 **Type of repair:** Direct suture: *n = *4 Mesh interposition: *n = *1 Recurrency: *n = *0
Takeda et al. [39]	Diaphragmatic Hernia Repair After Esophagectomy: Technical Report and Lessons After a Series of Cases	Retrospective, Monocentric 2009–2019	328 esophagectomies, 8 (2.4) patients were diagnosed with HH 18 months (7 days–39 months) after MIE and underwent hernia repair	Asymptomatic: *n = *1 Colon: *n = *4 Colon + small bowel: *n = *4 Emergency surgery: n/a Laparotomy: *n = *1 Laparoscopy: *n = *7 **Type of repair:** Suture repair: *n = *3 Mesh repair (n/a): *n = *5 Recurrency: *n = *1
Hanna et al. [17]	Hiatal Hernia after Esophagectomy: An Underappreciated Complication?	Retrospective, Monocentric, 2011–2017	258 of 310 esophagectomies analyzed, 79 (31%) patients had evidences of hiatal hernia, 44 of 79 had symptoms, 17 of 79 underwent hernia repair between < 1 and 39 months after index surgery	Asymptomatic: *n = *35 of all patients Colon: *n = *4 Colon + small bowel: *n = *4 Small bowel: *n = *9 Emergency surgery: *n = *8 Surgical approach: n/a **Type of repair:** Direct suture: *n = *14 Mesh repair: *n = *3 Recurrency: *n = *5
Fuchs et al. [10]	Transdiaphragmatic herniation after transthoracic esophagectomy: an underestimated problem	Retrospective, Monocentric, 2003–2017	39 patients underwent HH repair 259 (1–1467) days after hybrid Ivor Lewis esophagectomy	Asymptomatic: *n = *3 Colon: *n = *34 Small bowel: *n = *19 Emergency surgery: *n = *20 Laparotomy: *n = *29 Laparoscopy: *n = *10 **Type of repair:** Hiatoplasty: all patients (but, technique not described) Pexy of prolapsed organs to the diaphragm: *n = *10 Recurrency: *n = *3
Lubbers et al. [20]	Hiatal Hernia with Acute Obstructive Symptoms After Minimally Invasive Oesophagectomy	Retrospective, Monocentric, 2011–2018	307 total MIE (Ivor Lewis or McKeown) 8 (2.6%) patients underwent hernia repair 262 (1–1830) days after index surgery	Asymptomatic: *n = *0 Colon: *n = *3 Colon + small bowel: *n = *3 Small bowel: *n = *2 Emergency surgery: *n = *8 Laparotomy: *n = *3 (conversion: *n = *2) Laparoscopy: *n = *5 (additional thoracoscopy: *n = *1) **Type of repair:** Direct suture: *n = *5 Direct suture + mesh reinforcement: *n = *3 Recurrency: *n = *3

Terms used for the systematic review of English literature in *Medline* published from 01/2005–09/2020 reporting ≥ 5 surgical cases: “hiatal hernia” OR “hiatal herniation” OR “transdiaphragmatic herniation” OR “transdiaphragmatic hernia” OR “diaphragmatic hernia” OR “diaphragmatic herniation” OR “herniation” OR “hernia” OR “enterothorax” OR “cruroplasty” OR “hiatoplasty” AND “esophagectomy” OR “oesophagectomy” OR “gastrectomy”. * not further specified in the manuscript

*n/a* = not available in the manuscript, *HH* = hiatal hernia, *ILE *Ivor Lewis esophagectomy, *MI* minimally invasive, *POD* postoperative day. MIE = minimally invasive esophagectomy, *LAE* laparoscopically-assisted esophagectomy, *COS* conventional open surgery, *THE* trans-hiatal esophagectomy, *OE* open esophagectomy

Minimally-invasive esophago-gastric index surgery as a risk factor for trans-hiatal herniation due to decreased formation of abdominal adhesions, which has been repeatedly reported in the literature [11, 17, 19, 28, 30, 32, 33], seems to be underrepresented in the patient cohort of the present study. This might be due to the fact that the institutional standard for gastrectomy and trans-hiatal esophagectomy (with gastrectomy) was conventional open surgery and abdomino-thoracic esophagectomy was approached hybrid minimal-invasively in approximately one third of the patients in the past as published previously [5]. Nevertheless, minimally invasive surgery, especially for trans-hiatal and Ivor Lewis esophagectomy as well as structured surveillance with cross-sectional imaging are both increasingly provided to patients with esophago-gastric cancer. Furthermore, long-term survival is improved in upper gastrointestinal cancer patients due to better multimodal treatment strategies, thus trans-hiatal herniation after oncologic esophago-gastric surgery will become increasingly relevant in the future (note the rising number of reports in the more recent literature, Table 5) [11, 17, 19, 28, 30, 32]. However, evidences derived from the current literature or recommendations from medical societies clearing the questions on “how to approach the hiatus during esophago-gastric surgery “ and “when, why and how” to repair trans-hiatal herniation after esophagectomy or gastrectomy are not yet defined.

The most frequently prolapsed organ is transverse colon into the left chest (literature review in Table 5). Therefore, some risk factors for trans-hiatal hernia development after esophagectomy or gastrectomy can be found in the present patient cohort, including pre-existing hiatal hernia (which were present in three of five patients from the gastrectomy group), iatrogenic enlargement of the hiatus and diaphragmatic crus incision—especially of the left one, without resection, violence to the left pleura and higher tumor stages, especially of carcinoma of the esophago-gastric junction making crus resection necessary [7, 10, 11, 13–19, 21–23, 28–32, 34–37]. These lead consecutively to a loss of functional anatomy of the hiatus. Additionally, a lower BMI (< 25 kg/m^2^) or excessive weight loss after index surgery facilitates the mobility of intra-abdominal viscera and diabetes as well as neoadjuvant therapy impair wound healing [10, 11, 14, 17, 22, 30, 32, 36]. However, several measures have been described in the literature to restore hiatal function during index surgery in the hope to reduce the incidence of HH after esophago-gastric surgery, but data on their efficacy are still lacking: direct closure of the diaphragmatic defect anteriorly (because the conduit lies posteriorly) and/or posteriorly, fixation of the conduit to the crura, mesh interposition, colopexy or omentopexy in front of the hiatus [9–11, 13, 15–19, 34, 37, 38].

The most common symptoms of patients with trans-hiatal hernia after esophagectomy or gastrectomy are abdominal and/or thoracic pain as well as signs of bowel obstruction [7, 19]. The current literature agrees, that patients with severe and acute onset of symptoms should undergo urgent or emergency surgery for hernia repair [7, 10, 17]. Thereby, CT is an adequate technique in the elective as well as emergency situation for diagnosis of the hernia and assessment of possible hernia-associated complications of the prolapsed contents such as incarceration, strangulation or perforation [10, 11, 14, 30, 31, 36]. Fuchs et al. and Sutherland et al. described hernia-associated bowel complications in approximately one-third of the patients [10, 37]. This is relevant to know, as reflected by data of the present study reported in Table 2, if CT estimates ischemic bowel complications, rapid surgical therapy prevents patients from bowel resection (only necessary in one of seven patients with signs of hernia-associated vascular strangulation in preoperative CT). Approximately one-third of the affected patients develop post-esophagectomy HH early after index surgery, i.e. during the first 90 postoperative days due to lack of adhesions holding abdominal viscera in place [7, 8, 11, 19, 20, 28]. These patients suffered more frequently from severe, acute respiratory and cardiac symptoms compared with patients, who developed HH in the long-run after index surgery [7, 11, 14, 19, 21]. Both are reflected in the current study by the group of patients who underwent hernia repair without the use of meshes (Tables 1 + 2) as well as results of their large multicentric study by Gust and colleagues [7]. The latter reported high rates for emergency post-esophagectomy HH repair of being 76% during the first 90 days and 41% beyond the first year after esophagectomy [7]. Therefore, the indication for surgical correction of trans-hiatal hernia after esophago-gastric surgery in moderate to severe symptomatic patients remains undoubtedly, whereas, whether asymptomatic to mild symptomatic patients should undergo either elective hernia repair or clinical observation with a “wait-and-see” strategy is controversially discussed in the current literature (literature review in Table 5) [10, 14, 17, 20]. In the present study two of the patients, who underwent hernia repair, were asymptomatic. This goes in line with several reports in the current literature, which reported hernia repair even in patients without or with only mild symptoms, where the hernia had been found incidentally. This strategy follows the hypotheses, that the hernia may be progressive in size and symptoms, thus symptoms may arise in initially asymptomatic patients and an early elective hernia repair might prevent patients from severe cardiac, pulmonary and bowel complications as well as the fatal risk of incarceration with obstruction or perforation in the emergency situation [10, 15, 17, 30, 31]. By that way, no predictors are known for an unfavorable outcome of conservative “wait-and-see” strategies, thus a great proportion of patients require emergency surgery although an initially intended “wait-and-see” concept [14, 28, 30, 39]. Thereby, it is known that not only morbidity rates of the patients (up to 60%) with consecutively longer hospital stays but also mortality rates (up to 20%) are dramatically increased after hernia repair in the emergency situation [11, 14, 15, 20, 28, 29, 40].

The question on “how to sufficiently repair” trans-hiatal herniation after esophagectomy or gastrectomy cannot be adequately answered from case reports and small case series published in the literature. Surgical closure techniques depend on surgeons´ expertise and on characteristics of the hernia. Nearly all authors report a trans-abdominal approach for the hernia repair, with laparoscopy being safely feasible, especially in patients, who had undergone minimally-invasive approaches for index surgery, considering conversion rates up to 42% [7, 16, 28]. Vice versa, in emergency cases the preferred approach for hernia repair is conventional laparotomy [7, 14, 16]. However, surgeons should be prepared to undertake a thoracic approach, if dense intra-thoracic adhesions prevent the reduction of the prolapsed contents into the abdominal cavity [19, 21, 40, 41]. After reduction of prolapsed contents, essential steps during post-esophagectomy or post-gastrectomy trans-hiatal hernia repair are: assessment of the gastric conduit or jejunum to the anastomosis, assessment of herniated bowel viability, closure of the diaphragmatic defect around the conduit or jejunal limb, respectively, and thereby recreate a functional hiatus [18].

No consensus or broad experiences exit for the adequate repair technique resulting in extraordinary high recurrence rates reported in the literature. Surgeons have to be aware of different closure techniques of the hiatal orifice. However, our data show that the native size of the hernial orifice obtained from preoperative diagnostic imaging is not an adequate predictor of the appropriate closure technique especially regarding the intraoperative option for mobilization and reapproximation of the diaphragmatic crura followed by suture cruroplasty with or without mesh augmentation versus the need for tension-free mesh interposition (Table 2). In some cases anterior and posterior suture cruroplasty—the conduit lies dorsal –might be sufficient for hernia repair. Kent et al. recommend preservation of the peritoneal lining and dorsal mobilization of the crura for tension-free adaptation [40, 42]. Additional mesh augmentation can be performed after suture cruroplasty of larger defects similar to paraesophageal hernia repair [8, 16, 39, 43, 44]. Furthermore, some authors recommend the fixation of the conduit to the crus or pexy of abdominal organs, especially of the transverse colon as the most often prolapsed organ as an additional measure [10, 14, 16, 19, 32, 40]. Interestingly, the latter was done in our patient cohort more frequently in patients who underwent suture cruroplasty without the use of meshes. If tension is too strong for primary suturing, closure of the hiatal defect can be achieved by mesh as an inlay patch anteriorly from the conduit to the diaphragm with no approximation of the crural muscles [15, 23, 32, 40]. Nevertheless, most articles in the current literature report a mixture of these techniques for hernia repair resulting in high recurrence rates, up to 38%, which, however, might be independently from mesh buttressing of suture cruroplasty [17, 19, 20, 28, 42]. Thereby care has to be taken on the blood supply of the jejunal limb or the gastric conduit from the right gastroepiploic artery after gastrectomy or esophagectomy, respectively, and not to obstruct the passage, which both can increase the risk for conduit necrosis and anastomotic leakages especially in cases of hiatal hernia repair in the early phase after gastrectomy or esophagectomy [9–11, 13, 16–21, 34]. The use of meshes to restore the integrity of the hiatus during the index surgery is still disputed due to the additional feared risk of erosion of the conduit [9, 10, 13, 16–19, 34].

Limitations of the former case series describing treatment modalities and outcome of patients with post-esophagectomy or post-gastrectomy HH from the current literature are even reflected by the present study (Table 5). The present data analysis narratively reviews the institutional experience with the hernia repair, however, the current study was not designed to evaluate incidences of HH development after esophagectomy or gastrectomy. Thus, the retrospective character of the study, the missing information regarding incidences of HH development after index surgery and the lack of an institutional structured follow-up protocol after HH surgery might be the strongest limitation. The long observational period with a small sample size on the one hand, the lack of evidence-based standard protocols for post-esophagectomy or post-gastrectomy HH repair on the other hand have to be stated as limitations as well. Regarding the latter, it remains unclear, why patients underwent more frequently crural repair by simple suture without mesh usage in an emergency situation. This might be due to the fear of mesh-related complications by local peritonitis through initially incarcerated bowel or due to single surgeon´s preferences and even expertise in the emergency setting. Both might be prone to bias in the current data analysis. Finally, the inhomogeneous patient collective regarding index surgeries leads to some differences in hernia characteristics and thus should be interpreted as a minor limitation. Thereby, the current literature reveals the lowest incidence for trans-hiatal herniation after conventional gastrectomy [13, 28, 32, 36], since in these cases other types of internal hernia, like Peterson´s hernia or herniation through the jejunojejunostomy mesenteric defect after Roux-Y reconstruction, are more frequent [13, 36, 45–47]. Thus, the group of patients, who initially underwent total gastrectomy with Roux-Y reconstruction, seems a bit overrepresented in our cohort. However, principles of HH repair remain the same for post-gastrectomy as well as for post-esophagectomy HH. These factors surely limit the evidence regarding considerations of therapeutic strategies (emergency versus elective hernia repair versus “wait and see” concepts) as well as modalities of hernia repair surgery (primary suture cruroplasty versus primary suture cruroplasty with mesh augmentation versus tension-free mesh interposition) from the current study. However, firm conclusions should generally not be drawn from retrospective case series for clinical practice.

In conclusion, surgeons should be aware of trans-hiatal hernia, which is certainly an underestimated problem after esophagectomy or gastrectomy. Experiences with the disease and expertise in surgical repair techniques are low, even in high-volume centers for upper gastrointestinal surgery. Referring to high recurrence and morbidity rates, guidelines or expert recommendations are urgently needed, clearing the open questions regarding appropriate surveillance or the role of hernia repair in asymptomatic patients and principles of the adequate hernia repair technique. In our opinion, surgical repair should be offered to all patients with post-esophagectomy or post-gastrectomy trans-hiatal herniation. Patients with acute onset of especially pulmonary and cardiac symptoms, clinical signs of bowel obstruction or radiological signs of incarceration should undergo emergency surgery.
